# Evidence-Based Physical Therapy for Individuals with Rett Syndrome: A Systematic Review

**DOI:** 10.3390/brainsci10070410

**Published:** 2020-06-30

**Authors:** Marta Fonzo, Felice Sirico, Bruno Corrado

**Affiliations:** Department of Public Health, University of Naples “Federico II”, 80131 Naples, Italy; ma.fonzo05@gmail.com (M.F.); sirico.felice@gmail.com (F.S.)

**Keywords:** Rett syndrome, intellectual disability, movement disorders, physical therapy modalities, physiotherapy, rehabilitation, evidence-based medicine

## Abstract

Rett syndrome is a rare genetic disorder that affects brain development and causes severe mental and physical disability. This systematic review analyzes the most recent evidence concerning the role of physical therapy in the management of individuals with Rett syndrome. The review was carried out in accordance with the Preferred Reporting Items for Systematic Reviews and Meta-Analyses. A total of 17319 studies were found in the main scientific databases. Applying the inclusion/exclusion criteria, 22 studies were admitted to the final phase of the review. Level of evidence of the included studies was assessed using the Oxford Centre for Evidence-Based Medicine—Levels of Evidence guide. Nine approaches to physical therapy for patients with Rett syndrome were identified: applied behavior analysis, conductive education, environmental enrichment, traditional physiotherapy with or without aids, hydrotherapy, treadmill, music therapy, computerized systems, and sensory-based treatment. It has been reported that patients had clinically benefited from the analysed approaches despite the fact that they did not have strong research evidence. According to the results, a multimodal individualized physical therapy program should be regularly recommended to patients with Rett syndrome in order to preserve autonomy and to improve quality of life. However, more high-quality studies are needed to confirm these findings.

## 1. Introduction

Rett syndrome (RTT) is a rare, progressive neurodevelopmental disorder which mostly affects females; its prevalence is estimated between 1:10000 and 1:15000 [1]. RTT was first described in the medical literature by an Austrian physician named Andreas Rett in 1966 [2]. Nowadays, RTT is considered as a part of a spectrum of disease related to mutation of the methyl CpG binding protein 2 (MECP2) gene, which is located on the long arm (q) of the X chromosome (Xq28). The MECP2 gene codes for a protein that may downregulate the activity of many other genes. Therefore, mutations in MECP2 gene lead to defective epigenetic regulatory molecules [3,4]. The spectrum of MECP2-related phenotypes includes classic RTT, variant RTT, MECP2-related severe neonatal encephalopathy, and Psychosis, Pyramidal signs, Parkinsonism, and Macro-orchidism (PPM)-X syndrome [5,6]. Clinical feature of RTT patients is highly variable [7]. Development generally proceeds normally for about 6 to 18 months after birth; at this point, RTT patients enter a period of developmental stagnation which is followed by loss of previously acquired skills, such as hand movements and ability to communicate. Between 12 months and 4 years after birth, children develop autistic-like behaviors (i.e., lack of interest in social interaction and language regression), problems in general dynamic coordination (ataxia), and stereotypic hand movements, the last of which are considered a hallmark of the disease. During this time period, patients often develop breathing irregularities, such as temporary stopping of breathing (apnea) or hyperventilation syndrome, irregular sleep-wake rhythm, feeding and swallowing difficulties, seizures, and irritability [8,9,10,11]. After this period of rapid deterioration, neurological features stabilize, and some affected children may even show slight improvement in the ability of communicate. In the last stage of the disease, patients develop serious motor impairment, also due to the onset of muscle weakness, joint contractures, and spasticity. A variety of additional symptoms can occur in RTT patients including scoliosis, osteopenia, bowel dysmotility, functional megacolon, obesity, and esotropia [12,13,14,15,16].

Life expectancy for people with RTT depends on the age when symptoms first begin and their severity. Many patients live well into adulthood, although they may require constant care. A 2010 study by Kirby et al. showed that almost all girls with RTT reach the age of 10, with a more than 50 percent chance of reaching age 50 [17].

Symptoms, progression, and severity of people with RTT can vary from one patient to another, leading to a wide range of neuromotor and intellectual disabilities. The management of such disabilities requires a multidisciplinary approach. The RTT multidisciplinary team may include (1) medical and surgical subspecialists, such as pediatricians, neurologists, child neuropsychiatrists, gastroenterologists, physiatrists, and orthopedic surgeons, and (2) allied health professionals, such as dieticians, physiotherapists, speech and occupational therapists, psychologists, and specialized nurses. In addition, alternative and complementary therapies could also be part of the RTT multidisciplinary management program [18].

In accordance with the 2006 guidelines by Lotan, patients with RTT need an individually tailored intervention program for the entire duration of their life [19]. Such a program should be the result of a careful evaluation employed by the group of patient, medical doctors, caregivers and therapists. The intervention program originating from such an evaluation should create a continuous network of human support around the individual with RTT, in order to improve his quality of life by overcoming or reducing limitations.

Physical therapy is a branch of rehabilitation and its aim is to preserve, enhance, or restore movement and physical function impaired or threatened by disease, injury, or disability, using therapeutic exercise, physical modalities, assistive devices, and patient education and training. Physiotherapy is an essential tool in the management of several neuromuscular diseases and genetic disorders, with quite strong evidence of effectiveness [20,21,22,23,24]; its usefulness is even more evident if those pathologies affect children [25,26,27,28,29,30,31,32].

Aim of the present review was to find the current best evidence concerning the role of physical therapy in the management of people with RTT.

The Population, Intervention, Comparator, Outcome (PICO) method was used to formulate the clinical query according to the following parameters: (a) Population: RTT patients, (b) Intervention: physical therapy, (c) Comparator: no physical therapy, and (d) Outcome: improvement of functional outcome and quality of life [33].

## 2. Materials and Methods

This systematic review was performed in accordance with the Preferred Reporting Items for Systematic Reviews and Meta-Analyses (PRISMA) [34].

### 2.1. Study Eligibility Criteria and Report Eligibility Criteria

Letters, comments, editorials, conference proceedings, and practice guidelines were not considered for this systematic review. Studies were not limited to any particular design. The following study eligibility criteria were applied: (a) patients of any age affected by RTT; (b) physical therapy applied to at least a proportion of the patients; (c) adequate data provided; (d) no limits applied as to the minimum length of the follow-up. The criteria for the report were as follows: (a) written in the English language; (b) including already published data; and (c) including studies published from January 2000 up until December 2019.

### 2.2. Information Sources

For the purpose of identifying relevant studies, a systematic review of the literature was performed using the following databases: PubMed, Cochrane Library, PeDro, and Google Scholar. The literature search was conducted by two investigators independently.

### 2.3. Search Strategy

The following search terms were used: ‘Rett syndrome’, ‘physical therapy’, ‘physiotherapy’, ‘rehabilitation’.

### 2.4. Study Selection

Eligibility assessment of the selected studies was performed independently by two reviewers in an unblinded and standardised manner. All titles and abstracts were screened and ineligible articles were excluded. Then, the full text of the studies meeting the inclusion criteria were reviewed in detail by investigators.

### 2.5. Data Collection Process

Data from original articles were recorded on a data extraction form. The following data were extracted by one investigator and then crosschecked by the other: general information concerning the study (lead author and year of publication), study design, physical therapy approach, number of participants, assessment tool, follow-up duration, main results/findings, and level of evidence (grade of recommendation). Disagreements between the two reviewers on extraction of data were resolved by discussion; if no agreement could be reached, a third reviewer would be invited to make a decision.

### 2.6. Level of Evidence Assessment Process

Level of evidence was assessed independently by two of the investigators using the Oxford Centre for Evidence-Based Medicine (OCEBM)—Levels of Evidence guide [35]. Publication bias was not assessed because of the small number of selected studies.

## 3. Results

A total of 17,319 potentially relevant records emerged from the keywords searching in PubMed (*n* = 319), Cochrane Library (*n* = 0), PeDro (*n* = 0), and Google Scholar (*n* = 17000) databases. 16400 records were immediately removed as they were letters, comments, editorials, conference proceedings, and practice guidelines. Following this, a screening of the title and abstract of the 919 remaining studies was made, resulting in the elimination of types of publications different from what was stated in the inclusion criteria. Duplicates were also excluded. This second screening allowed an additional 869 results to be eliminated. Therefore, the full-text version of a total of 50 articles was assessed; 28 articles did not meet the inclusion criteria and were excluded. Eventually, a total of 22 studies were considered eligible. The selected results were classified as follows: 14 case reports, 2 case series, 2 multiple baseline studies, 1 single-case A-B-A-B design study, 1 single-case AB design study, 1 case-control study, and 1 modified individually randomized stepped wedge trial. From the detailed analysis of selected articles, nine approaches to physical therapy in patients with RTT were identified: (1) applied behavior analysis, (2) conductive education, (3) environmental enrichment, (4) traditional physical therapy with or without aids, (5) hydrotherapy, (6) treadmill, (7) music therapy, (8) computerized systems and (9) sensory-based treatment (Snoezelen).

**Figure 1:**
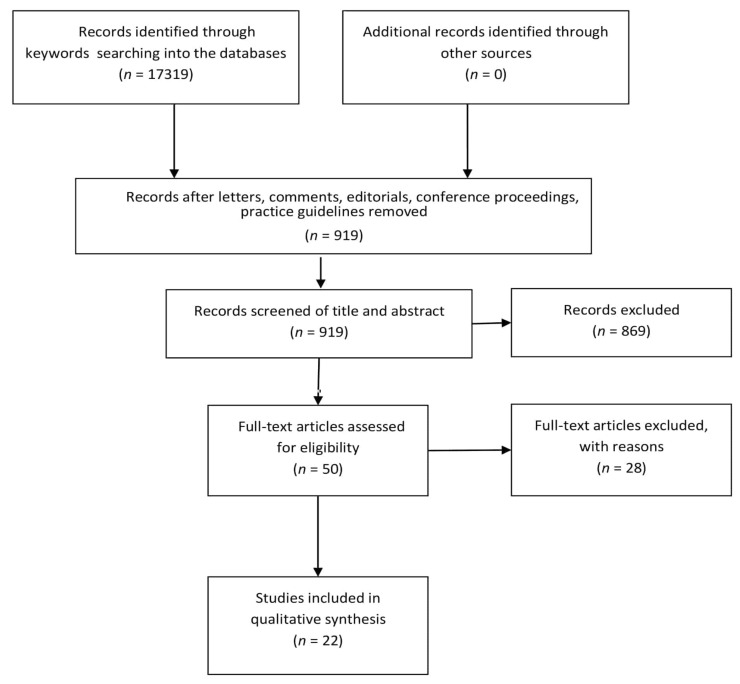
Preferred reporting items for systematic review and meta-analysis (PRISMA) flow chart concerning study retrieval and selection.

The PRISMA flow diagram used for study selection process is summarised in Figure 1. The details of studies selected for the systematic review are listed in Table 1.

### 3.1. Applied Behavior Analysis (ABA)

ABA was analyzed in two of the selected papers. In their 2007 review about alternative interventions for people affected by RTT, Lotan and colleagues stated that the use of ABA was extremely positive with reference to the acquisition of skills and the enhancement of the patient involvement in daily situations [43].

The 2015 study by Lotan et al. described a case report where ABA techniques succeeded in extending the patient daily walking ability [51].

### 3.2. Conductive Education (CE)

The 2012 single-case A-B design study by Lotan et al. assessed the functional skills of three girls with RTT aged 3–5 years before and during participation in a CE program [46]. Results showed that gross motor function skills improved at the end of the intervention period, but slightly declined in the following months of inactivity. 

### 3.3. Environmental Enrichment (EE)

The 2018 modified individually randomized stepped wedge trial by Downs and colleagues investigated the effects of EE on gross motor skills and Blood Brain Derived Neurotrophic Factor (BDNF) levels in a group of 12 girls with RTT [56]. EE was able to reduce functional deficit and to boost brain function after 6 months of treatment. Growth, sleep quality and mood were unaffected. 

### 3.4. Traditional Physical Therapy with or without Aids

Four of the selected papers belong to this group. In their 2001 case report, Larsson et al. stressed the importance of keeping the feet of RTT patients in good position making it possible to stand and walk. The combination of surgery, physiotherapy and well-fitting orthoses was used to manage feet malpositions [36]. The results showed that such combined approach allowed (1) to prevent muscle contracture and joint stiffness, (2) to regain ability to make transfers independently, and (3) to walk again even after a period of immobility.

The 2005 case report by Lotan et al. described a new management approach to scoliosis in a girl with RTT [41]. The patient underwent intensive therapeutic exercise, carried out with adapted equipment (cushion, seat, chair, and standing frame), and hydrotherapy. The results seemed to indicate that this intervention might be effective in treating scoliosis in RTT patients.

In their 2012 case report, Lotan and colleagues described a patient affected by RTT who regained her walking ability at the age of 28 years thanks to a three years of intensive activity programme, after being in a wheelchair for about five years [47]. The intervention programme included (1) a daily section implemented by the caregivers, consisting of lying position and walking exercises, and (2) a section performed by the physical therapist twice weekly, including joint mobility, balance training, walking exercises, and stair climbing and descending.

The 2015 case report by Schaefer-Campion et al. suggested that physical therapist and health professionals are essential in the process of selecting the assistive device for children with RTT to promote ambulation [52].

### 3.5. Hydrotherapy

In their 2003 case report, Bumin et al. investigated the effect of hydrotherapy, practiced in accordance to the Halliwick method for 8 weeks on an 11-year-old girl with RTT [38]. Immediately after hydrotherapy, stereotypical movements decreased, feeding activities and hand skills increased, walking balance was improved, interaction with environment intensified and hyperactive behavior and anxiety diminished. 

In the 2009 study by Lotan et al., the importance of water as a mediating environment for managing people with RTT was explained [45]. The authors introduced a case study of effective intervention strategies for a young child. Hydrotherapy improved communication and motor skills, enhancing the patient’s control over daily situations.

### 3.6. Treadmill

The 2004 study by Lotan et al. showed that a daily training program on a treadmill lasting two months was capable of improving functional ability in four girls with RTT [39]. Furthermore, the authors stated that non-professional personnel could execute such a program under supervision of a qualified physical therapist. This was a non-controlled observational clinical trial. 

The 2018 case-control study by Larsson et al. investigated the autonomic responses during walking on a treadmill in 12 females with RTT and in 14 healthy females [57]. The results showed that individuals with RTT could walk continuously for up to six minutes at their own maximum sustainable speed on a treadmill. The autonomic reactions in RTT patients differed only in the time course but not in amplitude compared with that in the control group.

### 3.7. Music Therapy

In the 2001 case report by Yasuhara et al., thirty-minute private sessions of active music therapy were used to treat three children with RTT. At the end of the treatment, patients showed (1) some degree of mental and physical development, (2) improvement of purposive hand use, and (3) development of language comprehension [37].

In their 2004 case report, Elefant and colleagues showed that a dual music and physical intervention approach allowed to shorten treatment time and to reach therapy possibilities that were unavailable with single therapeutic arrangement [40]. Patient’s communication choice-making abilities advanced dramatically, with positive effects on independence, self-esteem, self-confidence, and quality of life. 

The 2013 case report by Hackett et al. showed that six months of music therapy provided opportunities for a four-year-old child with RRT to increase skills related to functional hand use and social interaction [48].

### 3.8. Computerized Systems

The 2008 case report by Pizzamiglio et al. stated that an experimental, computerized visual-motor coordination training in association with a sensory-motor rehabilitative program based on Piaget’s theory, lasting 3 years, provided a patient with RTT with a partial recovery of voluntary and purposeful use of the hands. Such a program promoted the development of action and interaction schemes with the external world and enhanced behaviors suitable to the environment stimulations [44].

The 2013 multiple baseline design study by Stasolla et al. showed that a microswitch-based program lasting 6 months increased performance and indices of happiness and decreased stereotyped behaviors for two girls with RTT and multiple disabilities [49].

In their 2014 study, Lancioni et al. stated that microswitch-aided programs for adults with RTT (1) increased level of responding and stimulation input, (2) represented a very advantageous opportunity thanks to the use of activation responses, that could be performed through different movements, and (3) increased indices of happiness [50].

The 2015 study by Stasolla et al. showed that the use of assistive technologies was able to increase the adaptive responses and to decrease the stereotyped behaviors in three girls with RTT [53]. Moreover, during intervention patients improved their mood with positive consequences on the quality of life. In conclusion, the authors stated that assistive technologies based programs could be useful to increase occupation opportunities in people with RTT.

In their 2016 case series, Mraz and colleagues analyzed the feasibility of using an internet-based virtual reality intervention (FAAST software and Microsoft Kinetect sensor) for six girls with RTT [54]. The results showed decrease of hand stereotypes and increase of hand and arm movements away from the midline during intervention. Interviews and observation revealed successful game play when games were motivating, clearly established cause and effect, and matched level of cognitive ability of the participant. Furthermore, the authors stated that internet-based virtual reality intervention should be highly individualized to increase motivation and success of intervention.

The 2017 study by McAmis et al. showed that the virtual reality system named VR_Color, specifically tailored for people with RTT, was able to decrease the characteristic repetitive hand movements and to increase the use of hands in skilled functions [55]. Moreover, this intervention proved to be successful in severe RTT cases, differently from the FAAST system, which has not been tested in the treatment of such patients [54].

### 3.9. Sensory-Based Treatment (Snoezelen)

The 2006 article by Lotan reviewed the available scientific materials on the topic of Snoezelen, also known as controlled multisensory environment, incorporating clinical knowledge in the field of RTT and suggesting this approach as an appropriate intervention method for this population [42]. Three case reports have been analyzed; Snoezelen was able to relax the patient, to reduce muscle tone, to improve articular range of motion, and to enhance gait and balance. No posture improvement was reported.

## 4. Discussion

RTT is a rare, neurodevelopmental disorder that primarily affects females resulting in severe cognitive and physical disabilities. Treatment of RTT is purely supportive as there are no specific therapies currently available. RTT is associated with a high prevalence of comorbidities, the management of which requires a multidisciplinary approach. With multidisciplinary health care, people with RTT can enjoy a better quality of life and a considerably longer lifespan. In order to achieve such positive results, the RTT multidisciplinary team should always refer to guidelines that provide a practical and ethical framework for decision-making. At present, the only available guidelines concerning physical therapy in RTT are the 2006 guidelines for individual intervention by Lotan, the 2009 guidelines for management of scoliosis by Downs et al., and the 2016 guidelines for management of bone health by Jefferson et al. [14,19,58]. One of the essential requirements of a trustworthy guideline is that it should be based on systematic reviews of the best available evidence, and should include assessment of the quality of evidence, whereas all the above-mentioned guidelines for physical therapy in people with RTT were based only on expert opinions. This led to the need for carrying out this systematic review of the literature. 

This systematic review showed that available evidence for physiotherapy in RTT is low. The level of evidence of included studies ranged from 2b to 4 on the OCEBM scale. One study was a modified individually randomized stepped wedge trial with level of evidence 2b. One study was a case-control study with level of evidence 3b. The remaining studies were case reports and case series with level of evidence 4. Hence, in agreement with the OCEBM grades of recommendation, one of the selected studies was categorized as grade B and all the remaining studies as grade C (Table 1).

Regardless of the type of intervention practiced, all studies included in this review demonstrated that physical therapy improved the quality of life in patients diagnosed with RTT, mainly helping to preserve autonomy. On the other side, caution should be adopted in the interpretation of our findings since studies were highly heterogeneous in terms of participants, diagnostic criteria, interventions and outcomes. In particular, we would recommend the use of shared criteria in the diagnosis of RTT in order to make studies comparable [59].

A series of physical therapies are currently available to reduce or prevent limitations in people with RTT and to improve their quality of life. Such physiotherapies range from traditional approaches, like joint mobilization and hydrotherapy, to innovative methods, e.g. virtual reality and assistive technologies, and to almost alternative interventions, such as music therapy. Scientific literature showed that patients had clinically benefited from those different approaches despite the lack of strong research evidence. Several significant statements arose from this review: (1) early developmental intervention is imperative in order to assure that people with RTT reach their full potential, which is different for each patient; (2) physical therapy programs need to be individualized, both regarding types of intervention and program development, so that each patient may receive the most appropriate treatment for the stage of the disease and its personal needs, (3) currently no single reviewed physical therapy can be recommended over another for the lack of cross-comparative studies, but there is some level of evidence for each individual therapy with regards to its specifically measured outcomes, (4) multifaceted interventions lead to good results, and (5) a suitable intervention program must include appropriate involvement of the patient’s family or caregivers.

This is the first systematic review concerning physiotherapy in RTT. The present review was carried out following PRISMA reporting guidelines, with a clear, detailed and reproducible methodology.

This review has several limitations: (1) the small number of studies selected for the final phase, (2) the fair methodological quality of selected papers (e.g. small sample size, heterogeneity of interventions and assessment tools, short follow-up duration), (3) the impossibility of carrying out a meta-analysis, and (4) the lack of publication bias assessment. However, as stated in 2017 by Rath and colleagues, “clinical research in rare diseases needs to face several barriers that comprise the difficulty to recruit participants because of rarity, scattering of the patients, limited knowledge on natural history of diseases, difficulties to achieve accurate diagnosis and identify patients in health information systems, and difficulties choosing clinically relevant outcomes” [60].

Evidence-based physical therapy has been defined by Herbert as “physiotherapy informed by integration of relevant high-quality clinical research, patients’ preferences and physiotherapists’ practice knowledge” [61]. Nevertheless, in the event that high-quality clinical research is not available, like for RTT, Herbert stated that “good practice must make use of sources of any other lower quality research to inform action in practice” [61]. Therefore, we are confident that the findings of this systematic review will provide the best available practice management to clinicians and other health care professionals that work with RTT individuals every day.

## 5. Conclusions

A physical therapy program should be regularly recommended to patients with RTT, in order to preserve and restore movement and physical function threatened by the disease. This approach should be always individualized and adjusted to the needs of the patients. Preserving autonomy, improving quality of life, and supporting family caregivers should be the main objectives of such an approach.

Since the level of evidence for the scientific literature concerning the topic is currently low, future research should focus on carrying out studies with a better methodology and higher level of evidence. Given the difficulty in conducting traditional Randomized Controlled Trials (RTCs) on this topic, a valid alternative could be to perform Stepped Wedge Trials (SWTs), which involve a sequential roll-out of an intervention to participants over a number of time periods, such as in the 2018 study by Downs et al. [26].

## Figures and Tables

**Table 1:** Summary of the studies included in the review.

Lead Author and Year of Publication	Study Design	Physical Therapy Approach	Number of Participants	Assessment Tool	Follow-up Duration	Main Results/Findings	Level of Evidence (Grade of Recom- Mendation)
Larsson G. 2001 [36]	Case report	Kinesiotherapy + orthoses + aids (following surgery)	3	Narrative summary	Different for each case	– Regaining considerable walking was possible for a 36-year-old woman after 15 years in a wheelchair – Regaining transitional ability from floor to chair was possible when the clues to memory and motivation were found – Development of contractures of feet could be prevented in a 9-year-old – The ability to get up from the floor could be preserved in a 10-year-old girl	4 (C)
Yasuhara A. 2001 [37]	Case report	Music therapy (individual session of 30 min/week)	3	– A written description and videotape – The Denver developmental screening test (the Denver II) – The developmental psychology of music	40 weeks for two patients and 20 weeks for the third patient	The children showed some degree of mental and physical development: improvement of the purposive hand use, development of language comprehension, development of the ability to communicate by using cards or gestures, development in listening and playing instruments	4 (C)
Bumin G. 2003 [38]	Case report	Hydrotherapy (Halliwick method)	1	The tests included: – Analysis of stereotypical movements from a 5-minute video camera recording – Functional hand use according to performance in eating crackers placed on the table – Hand skills (grasping, holding, transferring small and large objects, finger feeding and drinking abilities) – Gait and balance – Hyperactive behaviour – Communication and social interaction	8 weeks	– Hand-to-mouth and hand-squeezing movements disappeared, while hand wringing movements appeared – The amount of stereotypical movements decreased – Feeding skills and hand skills in transferring objects and holding them improved – Walking balance improved – Interaction with the environment increased – Hyperactive behaviour and anxiety decreased	4 (C)
Lotan M. 2004 [39]	Case series	A daily training program on a treadmill	4	– Pulse measurement was used to evaluate aerobic physical condition at rest, the lowest pulse during 5 min of seating, and during training, the highest measurement during 5 min of walking at 1.5 km/h, with 0 inclination – Functional measurement was based on a 31-item scale specially established for the present study	2 months	– The average heart rates at rest were found to be 111.0, 109.0 and 89.0 at times 1, 2 and 3, while the average hearth rates during activity were found to be 145.3, 145.0 and 121.5 at time 1, 2 and 3. – Four items were found to have a significant change between measurement taken before and after intervention, namely knee walking, going up and down stairs, and walking speed for a distance of 25 m.	4 (C)
Elefant C. 2004 [40]	Case report	Dual intervention: physical and music therapy	1	Narrative summary	N.S.	– Treatment time was shortened – The difficulty treatment levels were raised – The joint treatment demands were more complex – Communication choice-making abilities advanced	4 (C)
Lotan M. 2005 [41]	Case report	A management plan consisting of: opposing asymmetry postures, walking and/or standing, maintaining spinal mobility through passive manual manipulation, individualized aids, parental and staff guidance	1	Measurement of the Cobb angle by X-ray in supine and suspended position	1 year and 6 months	– The Cobb angle in supine position switched from 29° to 20° – The Cobb angle in suspended position switched from 22° to 20°	4 (C)
Lotan M. 2006 [42]	Case report	Snoezelen (Controlled Multisensory Environment)	3 (case 1 stage II; case 2 stage III; case 3 stage IV)	Narrative summary	– Case 1: 3 months – case 2: 6 months – case 3: 4 months	Case 1: Agitation diminished and the patient was much more relaxed throughout the day Case 2: falls have completely ceased although posture did not seem to visually change Case 3: JROM increased throughout the body, enough to ease patient’s daily suffering and alleviate caregivers’ difficulties	4 (C)
Lotan M. 2007 [43]	Case report	Different alternative therapeutic interventions: Animal-Assisted Therapy (AAT), Auditory Integration Training (AIT), hyperbaric chamber, acupuncture/acupressure, aromatherapy, craniosacral therapy, Mayo fascial release, chiropractor, Reiki, Treager massage, cognitive rehabilitation, Applied Behavior Analysis (ABA), Advanced Biomechanical Rehabilitation (ABR), Doman-Delacato approach, Yoga	1	Narrative summary	3 years	The patient definitely made meaningful and significant progress for herself	4 (C)
Pizzamiglio M.R. 2008 [44]	Case report	Acclimating to the therapeutic setting + computerized visual-motor coordination training + sensory-motor rehabilitative program	1	– Bayley Scales of Infant Development (BSID-II) – Mental scale of the BSID-II – Uzgiris-Hunt Ordinal Scales of Psychological Development – MacArthur Communicative Development Inventory – Behavior Rating Scale (BRS) of the BSID-II	3 years	– BSID-II: the motor age switched from 11 months to 14 months – Mental scale of the BSID-II: the mental age switched from 4 months to 5 months. – Uzgiris–Hunt Ordinal Scales of Psychological Development: the total score switched from 3 to 32 – MacArthur Questionnaire (Sentences and Words): the total score switched from 2 to 56 – MacArthur Questionnaire (Gesturs): the total score switched from 1 to 11 – Behavior Rating Scale (BRS) of the BSID-II: the score switched from 67 to 145	4 (C)
Lotan M. 2009 [45]	Case report	Hydrotherapy	1	Narrative summary	3 years	The patient gained control over the body, thereby improving his functional abilities. Such gains, accompanied by improved communication skills, enhanced the child’s control over his daily situations, thereby achieving a feeling of self-worth and empowerment.	4 (C)
Lotan M. 2012 [46]	Single-case A-B design	Conductive Environment (CE)	3	– Rett Functional Evaluation Scale – Rett Syndrome Gross Motor Scale – Pediatric Evaluation of Disability Inventory (PEDI) – Hand Apraxia Scale.	2 years	– Rett Functional Evaluation Scale and Rett Syndrome Gross Motor Scale: improvement for all the patients. – PEDI: no changes. – Hand Apraxia Scale: hand function appeared poorer at the end of the intervention period, although this was not a clear trend because of the fluctuating assessment findings.	4 (C)
Lotan M. 2012 [47]	Case report	The intervention program includes two sections: 1. A daily section implemented by the staff which includes lying on the stomach and walking exercises. 2. A section performed by the physical therapist twice weekly which includes maintaining and enhancing the range of motion of the trunk and limbs, balance training, walking exercises, and stair climbing and descending.	1	Functional Independence Measure (FIM)	3 years	The FIM score switched from 18 to 25.	4 (C)
Hackett S. 2013 [48]	Case report	Music therapy	1	Retrospective video analysis	6 months	Hand movements have become more purposeful. Motor skills (specifically holding) enhanced. Intentional communication improved through promoting turn-tasking.	4 (C)
Stasolla F. 2013 [49]	Multiple Baseline	Microswitch-based program	2	– Microswitches activations independent of prompting – Means of percentage of intervals with indices of happiness – Means of percentage of intervals with stereotyped behavior	About 6 months	– Increased performance – increased indices of happiness – decreased stereotyped behaviors	4 (C)
Lancioni G.E. 2014 [50]	Single-case A-B-A-B	Microswitch-aided program (2 interventions)	1 with RTT and 1 with congenital encephalopathy	– Mean frequencies of microswitch responses per session – Mean percentages of observation intervals with indices of happiness	N.S.	Increase in microswitch responses and level of happiness for both participants during the intervention phases	4 (C)
Lotan M. 2015 [51]	Case report	Applied Behavioral Analysis (ABA)	1	Number of steps taken daily (accelerometer)	3 months	From 800 to 8000 steps/day	4 (C)
Schaefer- Campion C. 2015 [52]	Case report	A series of assistive device trials: – A metal toy shopping card – An anterior/posterior gait trainer with chest support – A posterior gait trainer with upper body supports and rotational handles – An anterior/posterior gait trainer with a seat, hip and chest supports – An anterior front-wheeled walker with lateral stationary handles – An anterior four-wheeled walker with a horizontal bar and lateral handholds	1	Gait analysis including: – Initiation of walking – The distance walked – Qualitative component	6 months	The anterior four-wheeled walker with a horizontal bar and lateral handholds was chosen	4 (C)
Stasolla F. 2015 [53]	Multiple baseline	Technological aids	3	(a) The number of objects inserted in the containers (b) The percentage of intervals with stereotypic behaviors (c) The percentage of intervals with indices of happiness	About 6 months (225 sessions)	(a) enhancement of the strategies of choice (b) reduction of stereotypic behaviors (c) beneficial effects on indices of happiness	4 (C)
Mraz K.M. 2016 [54]	Case series	Virtual Reality Intervention for Rett Syndrome (RTT-IVR)	6	Narrative summary + interviews	N.S.	Interviews and observation revealed successful game play when games were motivating, clearly established cause and effect, and matched level of cognitive ability of the participant	4 (C)
McAmis N.H. 2017 [55]	Case report	Virtual reality	1	System Usability Scale (SUS)	8 months	The ultimate feasibility percentile was calculated to be in the seventieth percentile which ranks in the “good” category.	4 (C)
Downs J. 2018 [56]	Modified individually randomized stepped wedge	Environmental Enrichment (EE)	12	– Rett Syndrome Gross Motor Scale (RSGMS) – Blood levels of BDNF – Body Mass Index (BMI) – Disorders of Initiating and Maintaining Sleep (DIMS) subscale of the parent-reported Sleep Disturbance Scale for Children – Rett Syndrome Behaviour Questionnaire (RSBQ)	6 months	– RSGMS: improvement – Blood levels of BDNF: improvement – BMI, DIMS, RSBQ: similar to baseline values	2b (B)
Larsson G. 2018 [57]	Case-control study	To walk on a treadmill at the maximum comfortable walking speed	12 RTT girls and 14 healthy females	– the NeuroScope^TM^ – A finger photopletismograph – Video-recordings	Six-minute single test	The changes in cardiac sensitivity to baroreflex and cardiac vagal tone in people with RTT compared to controls indicated more arousal, but only when the treadmill was started; as they continued walking, the arousal dropped to control level. People with RTT exhibited little changes in pCO2 whereas the controls showed increased values during walking.	3 (C)

(JROM)—joint range of motion, (RTT)—Rett syndrome, (BDNF)—blood brain derived neurotrophic factor.
